# Mosquito nets in a rural area of Western Kenya: ownership, use and quality

**DOI:** 10.1186/1475-2875-9-250

**Published:** 2010-09-02

**Authors:** Sophia Githinji, Susanne Herbst, Thomas Kistemann, Abdisalan M Noor

**Affiliations:** 1 Malaria Public Health and Epidemiology Group, Centre for Geographic Medicine, KEMRI-Wellcome Trust Collaborative Programme, Kenyatta National Hospital Grounds P.O. Box 43640-00100, Nairobi, Kenya; 2 Institute for Hygiene and Public Health, University of Bonn Sigmund-Freud-Str. 25 53105 Bonn, Germany; 3 Centre for Development Research, Department of Political and Cultural Change, Walter-Flex-Str. 3, 53113 Bonn, Germany; 4 Centre for Tropical Medicine, Nuffield Department of Clinical Medicine, University of Oxford, CCVTM, Oxford OX3 7LJ, UK

## Abstract

**Background:**

Insecticide-treated nets (ITNs) are regarded as one of the most effective strategies to prevent malaria in Africa. This study analyses the use and quality of nets owned by households in an area of high net coverage.

**Methods:**

A structured questionnaire on ownership and use of nets was administered to the households of individuals sampled from a local health centre in south Kisii district, Kenya. A physical inspection of all the nets in the households was done and their conditions recorded on spot check forms designed for that purpose.

**Results:**

Of the 670 households surveyed, 95% owned at least one net. Only 59% of household residents slept under a net during the night prior to the survey. 77% of those who slept under a net used an insecticide-treated net (ITN) or long-lasting insecticide-treated nets (LLIN). Out of 1,627 nets in the survey households, 40% were deemed to be of poor quality because of holes. Compared to other age groups, children aged 5-14 years were most likely to have slept under nets of poor quality (odds ratio 1.41; *p *= 0.007).

**Conclusions:**

Although net ownership was high following increased delivery of ITNs, continuous promotion of effective maintenance and routine use is needed and efforts to replace damaged nets must be implemented.

## Background

The widespread implementation of insecticide-treated nets (ITNs) is a major intervention strategy likely to significantly reduce morbidity and mortality from malaria across Africa [1]. The Kenya national programme for ITNs started in 2002 with social marketing that promoted subsidized nets within the existing retail sector. This was expanded to heavily subsidized nets provided to children and pregnant women through the maternal and child health clinics in 2004. Following a substantial grant from Global Fund to Fight AIDS, Tuberculosis and Malaria (GFATM), a campaign of mass distribution of free nets to all children younger than five years was organized in 2006. These measures have resulted in a rapid increase in ITN use by children aged less than five years from 7.1% in 2004 to 67.3% in 2006 in selected districts [2,3]. Monitoring ITN coverage in African countries is central to evaluating the progress of malaria control under the Roll Back Malaria (RBM) partnership [4]. Household possession of nets is an indicator of the extent to which distribution channels are enabling high coverage. Use of nets, however, is what affords protection and is therefore a more useful predictor of epidemiological impact [5]. Although increase in ITN coverage has been documented in several recent studies, [4,6-9] relatively little is reported on physical condition and intra-household net use. The objective of this paper is to evaluate the use and quality of nets in a rural setting where massive malaria control campaigns have been carried out.

## Methods

### Study area

The study was conducted in Nyamarambe division of south Kisii district, located in the malaria epidemic zone of the western highlands of Kenya. Malaria in the area is characterized by year round transmission with seasonal peaks following the heavy rains. Over the years, the area has received numerous malaria control interventions because of its susceptibility to epidemics.

### Sample population and survey procedures

Study participants were randomly sampled from patients who attended a local health care facility (Nduru health centre) during a high malaria transmission season (May to July) in 2007. A study clerk explained the survey to the patients or their parents/guardians as they were registered at the health centre. A written informed consent to participate in the study was then sought. Those who consented were listed and asked to provide details of their residence for follow-up in case they were sampled. Random numbers were used to select participants from the consenting individuals on a weekly basis. The households of those sampled were visited in the week following the subjects' attendance at the health facility. A standardized household questionnaire and a net spot check form were used to collect data. The questionnaire recorded data on ownership, number, source, cost, and insecticide treatment status of all bed nets in the households. Ownership was defined as having at least one net whether treated or not. Any net distributed through the free mass campaign was classified as treated as these were long-lasting insecticide-treated nets (LLIN). The treatment status of nets obtained from other sources was enquired from the respondents. The spot check form was designed to record data on the characteristics (colour, brand, and shape) of each net; the condition of the net (clean or dirty, holes or no holes, net hanging around the sleeping place or not), and whether the net was used the previous night. A net was classified as having holes if it had any finger-sized hole or larger. Net use was determined by asking which individuals in the household slept under a net during the night prior to the survey. A net was classified as unused if no member of the household slept under it during the night preceding the survey. Of those that were unused, reserve nets were defined as nets that had not been opened from the manufacturers' packets.

Key variables investigated were persons who slept under a bed net the night before the survey and the physical condition of the nets used. Net users were classified into five person types by age: children under 5, older children (5-14), women of reproductive age (15-49), adult males (15-49) and individuals aged 50 years and above. The independent variables used were: person type, number of people sleeping under the same net, number of reserve nets, net treated or not, source of net and socioeconomic status. Principal Component Analysis (PCA) was used to derive a socioeconomic index based on ownership of land, cash crops grown, domestic animals, availability of enough food supplies and a proxy of 14 durable household goods. Using the factor scores from the first principal component as weights, a household socio-economic score variable was constructed. The scores were used to classify the households into three broad socioeconomic groups; least poor, middle poor and most poor.

Association between the physical condition of the net and net user was evaluated for 301 households for which these data were available. These were the households interviewed after the spot check form was modified to include the names of specific household members using each net. This modification was made following a supervisory visit during the data collection exercise.

### Data analysis

Multivariate logistic regression analysis was undertaken to assess which person types were more likely to use the most protective nets. The dependent variable was net quality classified as 1 for intact nets and 0 for nets with holes. For comparison purposes, a similar model was developed using net treatment status (coded as 1 for treated and 0 for untreated nets) as the dependent variable.

### Ethical Approval

This study was approved by the government of Kenya, reference number: MOST 13/001/28C 66. A written informed consent was obtained from all the study participants or their respective caretakers.

## Results

The survey covered 670 households with 3,667 individuals and 1,627 nets. 95% of the surveyed households owned at least one net. 1,268 (78%) of the nets in the households were either ITNs or LLIN. The mean number of nets (treated and untreated) owned per household was 2.4. About 47% of the households obtained all their nets during the Ministry of Health mounted free mass distribution campaign in July 2006. 32% of households had non-campaign nets purchased from different sources while 18% of the households had both nets distributed during the free campaigns and non-campaign nets. Of the 317 households that owned non-campaign nets, 180 (57%) bought them at KES 50 while 64 (20%) purchased them at KES 100 (Table 1).

**Table 1:** Net ownership and source

*Ownership*	*n(%)*
Household owning nets	638(95.2)

Households without nets	32(4.8)

Total	670(100)

	

*No. of bed nets*	*No. of HH(%)*

0	32(4.8)

1	120(17.9)

2	244(36.4)

3	147(21.9)

4	88(13.1)

5+	39(5.8)

Total	670(100)

*Mean number of nets per HH 2.4*	

	

*Source of bed nets*	*No. of HH(%)*

Campaign nets	298(46.7)

Non campaign nets	205(32.1)

Campaign &non campaign nets	112(17.6)

*Other sources	23(3.6)

Total	638(100)

Cost of non-campaign nets (KES)	

50	180(56.8)

100	64(20.2)

> 100	73(23.0)

Total HH	317(100)

* 13 households got nets from friends/relatives, 10 households got nets from NGOs

### Net use

2,156 (59%) of people residing in the survey households slept under a net during the night preceding the survey. 59% of children under five and 58% of women in the reproductive age (15-49) slept under a net during the night prior to the survey. Among the adult men, 60% slept under a net the night prior to the survey (Table 2). 1391 (85%) of the nets were used during the night prior to the survey, giving a ratio of 1.5 persons per net. 236 (15%) of nets were not used. 88 (37%) of these unused nets had not been opened from the manufacturers' packet; 55 (63%) of the non opened nets were provided during the free campaigns.

**Table 2:** Previous night net users by person type

Characteristic	All residents	Nets users (%)
Females	1879	1095(58.3)

Males	1788	1061(59.3)

Total No. of HH members	3667	2156(58.8)

		

*Person type by age*		

Under 5	745	441(59.2)

Children (5-14)	908	530(58.4)

Women (15-49)	933	545(58.4)

Adult men (15-49)	806	487(60.4)

50 and above	258	150(58.1)

* Ages of 7 females and 10 males could not be established

### Condition of nets

**Figure 1:**
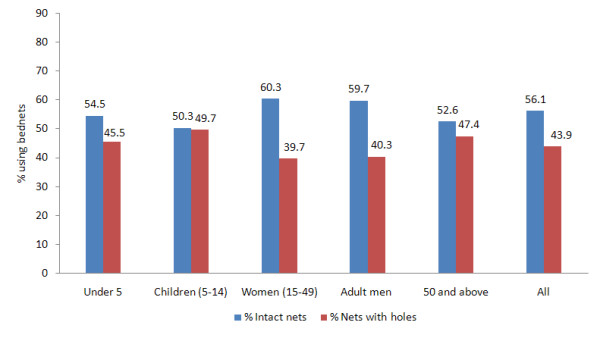
**Percentage of net users by age category sleeping under intact nets or nets with holes (N = 1527)**.

**Figure 2:**
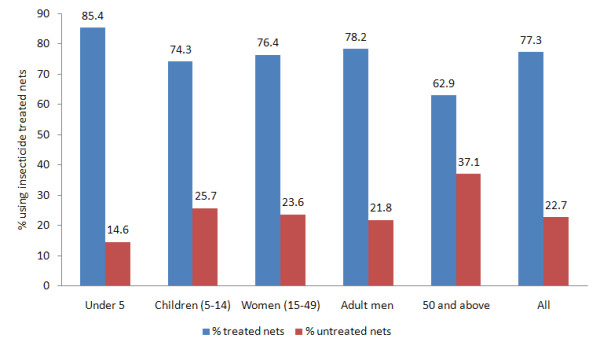
**Percentage of net users by age category sleeping under treated nets and untreated nets (N = 1527)**.

648 (40%) of the nets had holes; 318 (49%) of these damaged nets had been obtained through the free mass distribution campaigns between 9 and 12 months prior to the survey. 259 (40%) of the nets with holes were non-campaign nets mainly bought at subsidized prices over the two years preceding the survey. The source of 71 (11%) of the nets with holes could not be distinguished as the households had both campaign and non-campaign nets. A total of 359 (22%) nets requiring treatment were recorded in 127 (19%) of the survey households (Table 3). In the subset of data for which associations between the net quality and the net user were available, 44% of the household members slept under nets with holes. 50% of older children (5-14 years) slept under nets with holes while 60% of women aged 15-49 and adult men slept under intact nets (Figure 1). 77% of net users slept under a treated net (ITN or LLIN). 85% of children under five slept under treated nets (Figure 2).

**Table 3:** Condition of bed nets in the households

*Net has holes*	*n(%)*
Yes	648(39.8)

No	979(60.2)

Total	1627(100)

	

*Source of nets with holes*	

Campaign nets	318(49.0)

Non campaign nets	259(40.0)

Not distinguished	71(11.0)

Total	648(100)

	

*Net treated*	

Yes	1268(77.9)

No	359(22.1)

Total	1627(100)

	

*Net hanging*	

Yes	1140(70.1)

No	487(29.9)

### Multivariate analysis: variables significantly associated with the physical condition of the net used

The multivariate logistic regression analysis suggested that children aged 5-14 were more likely to have slept under a net with holes compared to the other person types (odds ratio 1.41; *p *= 0.007). Similarly, individuals from the most poor households were more likely to sleep under nets with holes compared to the least poor (odds ratio 1.41; *p *= 0.005). Those who owned reserve nets were less likely to sleep under damaged nets (odds ratio 0.67; p = 0.000) compared to those who did not have reserve nets. Treated nets were less likely to have holes compared to un-treated ones (Table 4). Similar trends in results were observed in the model using net treatment status as the dependent variable.

**Table 4:** Multivariate analysis of factors significantly associated with net condition

Variable	Odds ratio	*p*-value	95% CI
Children 5-14	1.41	0.007	1.10 1.81

Most poor	1.41	0.005	1.11 1.79

Least poor	0.66	0.003	0.51 0.87

Net treated	0.74	0.017	0.58 0.95

Number of reserve nets	0.67	0.000	0.55 0.82

## Discussion

This study showed a relatively moderate proportion of net use among household residents (59%) despite a high coverage of 95% households owning at least one net. An assessment of 15 national surveys across Africa showed similar disparities between household possession of at least one ITN and previous night ITN use among children aged below 5 years and pregnant women [10]. Although the Kenya free mass distribution of long lasting insecticide treated nets (LLIN) has substantially increased household ownership of any net [3,11], the Kenya National Malaria Strategy (KNMS), which aims at 100% coverage with LLIN and 80% use in each targeted area, recognizes the need to bridge the persistent gap between ownership and use of nets [12].

Net use patterns were similar across the different age groups. This is unlike other studies [13-16] that have shown higher net use among children under 5. This suggests that non targeted household members are likely to benefit from nets distributed to the high risk groups. However, children under five and women of reproductive age in the current study represented the highest proportion of persons using treated nets. Similar findings were reported in Tanzania where infants and other vulnerable groups were most likely to sleep under the most protective nets [17]. The present study also showed that children aged between five and 14 were most likely to use damaged nets. Across Africa, the age group 5-19 has the highest proportion of those not protected by ITNs [18]. With declining transmission, it is expected that the burden of malaria will shift to older age groups and future net campaigns may therefore need to target older children with school-based distribution as a possible approach.

Spot-checks conducted in this survey revealed the poor physical condition of 40% of the nets within less than one year since distribution. Similar observations have been reported in Burundi, where despite a high rate of retention of LLINs distributed among targeted households, their lifespan and fabric quality was reported to have decreased quickly after the first year, mostly because they had developed holes [19]. Likewise, a net survey in Tanzania revealed that 44.9% of the nets had holes [20]. Our observations during the data collection exercise pointed to two major causes of holes: the wooden sticks used to support the nets around the sleeping areas and commonly used open tin lamps that burnt the bed nets. Although experimental studies have reported that purposely perforated ITNs can still kill mosquitoes and prevent mosquito bites, formation of holes is concurrent with insecticide loss. It is therefore important to take into account both aspects in determining the useful life of the nets [21,22]. The KNMS [12] states that LLINs will be distributed to all households in endemic and epidemic areas every three years. Data collected in this study shows that the physical quality and resilience of nets used in poor rural settings, may not last the three years planned in the government strategy, let alone the five years lifespan of the currently promoted long lasting insecticide treated nets. The physical condition of the nets was found to be associated with socioeconomic status. Although the scaling up of ITNs has been found to be associated with greater social equity in ownership [3], the socioeconomic status may influence the ability to take better care of the nets.

During the free net distribution campaigns, each child in the targeted age group got a net. This may have lead to accumulation of 'reserve nets' in households with more children aged less than five. A study in Ethiopia showed similar results where some ITNs had never been used and purchased nets were more likely to be used as compared to free nets [23]. Presence of surplus nets in the household may lead to abuse. Use of ITNs for drying fish, for example, has been reported among the fishing communities along lake Victoria where 84.5% of the nets used for that purpose were obtained for free or at subsidized prices [24].

There are a number of limitations in this study. The sample was drawn from the catchment area of just one health facility in a highland area of seasonal peak malaria transmission. This makes it difficult to generalize the results to other areas as the gap between net ownership and use may be different in perennial transmission zones. Unlike in other studies of this kind, the number of holes in the nets was not counted and their sizes were not measured beyond fitting a finger. Larger community based studies are required to evaluate net use patterns across different malaria epidemiological zones while taking into account the degree of damage of the nets.

## Conclusions

The findings of this study suggest the need of enhancing effective use and maintenance of nets in order to realize their full potential impact on malaria prevention. A more focussed distribution taking into account pre-existing nets and replacement of the ones that are badly worn out is required. As the Roll Back Malaria aims to reach 80% ITNs coverage among vulnerable groups by 2010 [25] the benefits acquired through scaling up ITNs coverage may be lost if the ITNs are either so worn out or not used effectively.

## Competing interests

The authors declare that they have no competing interests.

## Authors' contributions

SG designed, collected and analysed the data and drafted the manuscript. SH supervised the study and contributed to the revised versions of the manuscript. TK supervised the data management and analysis and contributed to the revised versions of the manuscript. AMN conceived the questions for this manuscript and provided direction on analysis and drafting the manuscript. All authors read and approved the final manuscript.
